# Cutaneous Eruptions Due to Bromide of Potassium

**Published:** 1870-02

**Authors:** 


### Cutaneous Eruptions due to Bromide of Potassium.

M. Vossin has observed in 96 cases of epilepsy treated with bromide of potassium, five different kinds of cutaneous eruption, which he imputes to the action of the drug, viz., (1) *Acne*, which is the most common, is preceded by itching; mostly affects the face and chest; occurs most frequently in full-blooded persons; and is unaffected by the season. The use of diuretics, together with a lotion of flax-seed tea, was found to keep the eruption in abeyance, without the need of suspending the bromide. (2) A *peculiar eruption* which occurred in six cases, and consisted of little tumors formed by groups of very indolent ance-like pustules, generally seated on the legs; inflamed at the base, and depressed at the centre; painful to the touch (except at the centre); tardily discharging a matter like that of furuncles; healing slowly, and leaving cicatrices which, when seated over a bone, are painful on pressure. They occur oftenest in the winter, and are accompanied by acne over the body. Early incision was of no benefit; but rest, and the application of poultices and opiated cerate, are recommended. (3) A variety of *urticaria*, occurring in two instances, and somewhat resembling erythema nodosum. It occurred only after long continuance of the bromide in large doses. (4) *Furuncles*. (5) A very moist *eczema* of the legs, with pityriasis of the scalp.—*Gazette des Hopitaux*, 1868.

